# The time has come to make cervical cancer prevention an essential part of comprehensive sexual and reproductive health services for HIV-positive women in low-income countries

**DOI:** 10.7448/IAS.18.6.20282

**Published:** 2015-12-01

**Authors:** Megan J Huchko, May Maloba, Miriam Nakalembe, Craig R Cohen

**Affiliations:** 1 Department of Obstetrics, Gynecology and Reproductive Sciences, University of California San Francisco, San Francisco, CA, USA; 2 Family AIDS Care and Education Services, Kenya Medical Research Institute, Kisumu, Kenya; 3 Department of Obstetrics and Gynaecology and Infectious Disease Institute, Makerere University, Kampala, Uganda

**Keywords:** cervical cancer prevention, HIV, integration, low- and middle-income countries

## Abstract

**Introduction:**

HIV and cervical cancer are intersecting epidemics that disproportionately affect one of the most vulnerable populations in the world: women in low- and middle-income countries (LMICs). Historically, the disparity in cervical cancer risk for women in LMICs has been due to the lack of organized screening and prevention programmes. In recent years, this risk has been augmented by the severity of the HIV epidemic in LMICs. HIV-positive women are at increased risk for developing cervical precancer and cancer, and while the introduction of antiretroviral therapy has dramatically improved life expectancies among HIV-positive women it has not been shown to improve cancer-related outcomes. Therefore, an increasing number of HIV-positive women are living in LMICs with limited or no access to cervical cancer screening programmes. In this commentary, we describe the gaps in cervical cancer prevention, the state of evidence for integrating cervical cancer prevention into HIV programmes and future directions for programme implementation and research.

**Discussion:**

Despite the biologic, behavioural and demographic overlap between HIV and cervical cancer, cervical cancer prevention has for the most part been left out of sexual and reproductive health (SRH) services for HIV-positive women. Lower cost primary and secondary prevention strategies for cervical cancer are becoming more widely available in LMICs, with increasing evidence for their efficacy and cost-effectiveness. Going forward, cervical cancer prevention must be considered a part of the essential package of SRH services for HIV-positive women. Effective cervical cancer prevention programmes will require a coordinated response from international policymakers and funders, national governments and community leaders. Leveraging the improvements in healthcare infrastructure created by the response to the global HIV epidemic through integration of services may be an effective way to make an impact to prevent cervical cancer among HIV-positive women, but more work remains to determine optimal approaches.

**Conclusions:**

Cervical cancer prevention is an essential part of comprehensive HIV care. In order to ensure maximal impact and cost-effectiveness, implementation strategies for screening programmes must be adapted and rigorously evaluated through a framework that includes equal participation with policymakers, programme planners and key stakeholders in the target communities.

## Introduction

The combined threat that cervical cancer and HIV present to women's quality of life, reproductive capacity and overall mortality highlights a glaring inequality in global women's health. The enormous international disparity in the incidence of and survival from cervical cancer has historically aligned most closely with country income [1]. Nearly 85% of cases and 87% of deaths occur in less developed regions, where several factors conspire to make cervical cancer a leading cause of cancer and cancer-related mortality [2]. Inadequate healthcare and public health infrastructure, competing health priorities and persistent poverty prevent large-scale cervical cancer prevention programmes from gaining traction, resulting in only a small minority of the population being screened [3]. Rates of cervical cancer in less developed countries are similar to those seen in the United States prior to the introduction of widespread screening [4].

In the last 20 years, the increased burden of cervical cancer has been intensified by the contribution of HIV to cervical precancer and cancer. Global maps showing country-specific HIV prevalence match the global maps of cervical cancer incidence and mortality. For example, the incidence of cervical cancer is 42.7 per 100,000 women in Eastern Africa, a high HIV-prevalence region with low screening coverage, compared to 30.6/100,000 in Middle Africa (moderate HIV prevalence, low screening) and 6.6/100,000 in Northern Africa (low HIV prevalence, moderate screening coverage) [5]. The high rate of HIV infection in many low- and middle-income countries (LMICs) has potentiated the already increased risk for cervical cancer for women living in these countries. The decrease in cellular immunity caused by HIV increases the risk for new and persistent human papillomavirus (HPV) infections – the primary cause of cervical cancers and precancerous cervical lesions – and contributes to an accelerated incidence and progression of cervical neoplasia [6, 7].

Increased availability of HIV care and treatment, combined with greater coverage of antiretroviral therapy (ART) in recent years, has been lifesaving for entire populations of HIV-positive women. In contrast to other AIDS-related malignancies, which show improvement with ART, the positive effect of ART on cervical cancer outcomes is not clear [8–11]. Conversely, researchers have shown that the risk of anal cancer, another HPV-related malignancy, actually increases after ART use, making it plausible that the biologic risk for cervical cancer may increase [12]. Regardless of the direct biologic effect of ART on cervical cancer risk, in the many LMICs that have addressed their high HIV prevalence through improved HIV testing and access to treatment, there is a significant increase in the number of HIV-positive women living longer with excess cervical cancer risk [13]. This makes the implementation of effective screening programmes an urgent public health priority, especially for the HIV-positive women who are most vulnerable to the disease.

There is a precedent of successful partnerships between international donors and local governments to strengthen healthcare infrastructure and build local capacity in ways that helped to stem the HIV epidemic. Many government health systems have successfully leveraged these gains in the healthcare system and numbers of trained healthcare workers to address other healthcare needs such as tuberculosis, malaria, family planning, maternal health and other non-communicable diseases (NCDs) [14–16]. As evidence for the efficacy and cost-effectiveness of integrating these other health services increases, there has been an increase in donor funding and policy commitment to support integration. However, cervical cancer is routinely excluded from the definition of *sexual and reproductive health* (SRH) *services*, which often focus on family planning, prevention of maternal-to-child transmission (PMTCT) of HIV and sexually transmitted infection (STI) prevention [17–20]. While the World Health Organization (WHO) *2006 Guidelines on Sexual and Reproductive Health for Women Living with HIV* do include cervical cancer screening as a topic area, inclusion of cervical cancer prevention as part of essential services for HIV-positive women is not a focus of that document. Rather, the section on cervical cancer concludes with recommendations for HIV-positive women to have the same access to cervical cancer screening as HIV-negative women [21]. As research into integration of reproductive health and HIV services evolved, more recent documents that focus on provision of comprehensive care for people living with HIV in LMICs include recommendations on how to integrate family planning, STI prevention and PMTCT, while cervical cancer is not mentioned [22, 23]. The global health community is failing women in a crucial way: it has neglected prevention, screening and treatment for cervical cancer among the highest risk population, HIV-positive women in LMICs. In this commentary, we describe the current policy and evidence around strategies for implementing cervical cancer into HIV care and recommend future research and policy directions to ensure that cervical cancer prevention is included as part of essential SRH services for HIV-positive women.

## Discussion

There are several reasons for the exclusion of cervical cancer as part of comprehensive care for women living with HIV. Primarily, the majority of the world's HIV-positive women live in countries where there is no access to cervical cancer prevention for anyone, regardless of their serostatus. One of the effects of this lack of screening infrastructure is an absence of cancer registries in most LMICs. Without accurate estimates of the number of cases each year and the impact of HIV on the incidence and prevalence of cervical cancer, it is impossible to set targets and track progress in addressing this issue. Another reason for the exclusion of cervical cancer from SRH services offered to HIV-positive women is that, despite being caused by an infectious agent, it is often conceptualized as an NCD, rather than a component of SRH. Instead of receiving increased attention by having a home in two different content areas, this dual identity has actually led to less focus and attention for cervical cancer prevention, which is often seen as not fully belonging in either category. As the immediate and pressing needs of the HIV epidemic have begun to abate, there is an opportunity to use the lessons from both NCD and SRH management to address cervical cancer prevention in a way that best fits the unique characteristics of the disease.

Cervical cancer prevention fits into an NCD paradigm of integrating preventative care into existing clinics through periodic, evidence-based screening, with treatment of early or preclinical disease. Importantly, though, because of the counselling, outreach, screening techniques and fertility implications for treatment of invasive disease, cervical cancer prevention has a natural place in SRH services. Providers who are more comfortable talking to women about their reproductive health and family planning, and who can ably counsel women and perform pelvic exams, may be better suited to perform cervical cancer screening [24]. A successful cervical cancer prevention programme should include elements from NCD prevention strategies (disease awareness, coupled with periodic, universal screening and access to risk-reduction interventions) when providing services under the paradigm of reproductive health.

Another key reason for the exclusion of cervical cancer from primary healthcare in LMICs, and more recently from comprehensive HIV care, was the lack of feasible and affordable prevention strategies. We now have a wide range of low-cost and effective primary and secondary prevention options that can be operationalized in LMICs, making dramatic global reductions in cervical cancer incidence a realistic goal within a generation. HPV vaccination is the most successful and cost-effective strategy for cervical cancer prevention, especially in high HIV-prevalence areas [25–27]. The WHO has prequalified two HPV vaccines that could dramatically reduce cervical cancer deaths in LMICs if vaccination coverage can be scaled up [28]. GAVI, the Global Vaccine Alliance, is supporting initiatives to provide vaccines in selected LMICs, and pilot delivery programmes are ongoing [29]. The vaccination of adolescent girls also provides an opportunity to provide them with other reproductive health services and health education (including family planning and menstrual hygiene); it would provide primary prevention of HPV and cervical cancer prior to sexual exposure to HIV. Ensuring that adolescent girls have the opportunity to receive a vaccine that protects them from the morbidity and mortality related to cervical cancer should be a key global health priority.

Conventional screening methods, using Pap smears and biopsies, require infrastructure and clinical expertise and are hard to scale up in LMICs. However simpler, cheaper screening techniques, such as visual inspection with acetic acid (VIA) and HPV DNA testing, hold great promise and are undergoing widespread evaluation [30, 31]. The WHO Global Action Plan on NCDs describes screening with VIA as a “best buy,” meaning that it is both highly cost effective (i.e. it costs less than the per capita gross domestic product to avert one disability adjusted life-year) and it is feasible to implement in settings with constrained health systems [32]. There are promising results from large trials, suggesting that VIA can reduce cervical cancer incidence by 25 to 30% [33], with similar performance characteristics among HIV-positive women compared to HIV-negative women [34]. Although screening with HPV is more expensive than with VIA, a study by Goldie *et al*. [35] in five LMICs found that HPV screening is very cost-effective, and a single test at age 35 years reduces lifetime cancer risk by 25 to 36%. This finding has been supported in models of HPV screening among HIV-positive women [36]. Ongoing and completed studies are looking at novel strategies to maximize uptake of HPV screening, including self-collection and community health campaign models, in low-resource/high HIV-prevalence settings [37, 38]. The WHO has recognized and summarized the evidence for low-cost cervical cancer prevention strategies in their *2013 Comprehensive Cervical Cancer Prevention and Control Manual* [39], which includes recommendations for screening strategies for HIV-positive women.

One strategy for ensuring that HIV-positive women access cervical cancer screening and prevention is through service integration. Integrating care for HIV, sexual health, reproductive health and maternal health has been shown to improve uptake of services, reduce HIV-related stigma and improve the quality of care received by women [40, 41]. Although there are many definitions of *integration*, the model that is most feasible for cervical cancer and HIV care is integration of cervical cancer services into existing HIV-care programmes, given the lack of standalone cervical cancer prevention clinics and periodicity of screening. There is growing evidence for the feasibility of integrating cervical cancer prevention into HIV services using low-cost screening strategies coupled with treatment for precancerous lesions [42–45]. Furthermore, integrating cervical cancer prevention services into HIV primary-care facilities, rather than referring women to a separate family planning or reproductive health facility, provides an opportunity to include and educate male partners, which may be particularly important in areas where men have control over healthcare decisions [46, 47].

However, integration may not be feasible or successful in all settings. While integration holds the promise of leveraging stronger health systems to improve access to and uptake of secondary services in higher risk populations through a decrease in the visit burden and loss to follow-up, several studies in sub-Saharan Africa have shown significant weaknesses in models of various health services integrated into HIV care. These include limited interest among the general population in receiving care through integrated models [41], concerns about disclosure and resultant stigma in general outpatient settings [40], lack of clear policies, unacceptable clinical load on the staff, longer wait times and concerns about quality of care [48].

While the promise of integration has not been borne out in every setting, this does not mean that it should be discarded for the next big idea in service delivery. One randomized study of integrated HIV and antenatal services showed high rates of attrition in both arms, suggesting that there are structural barriers to uptake that lie outside of the care model [49]. This finding, along with the difficulties experienced in different settings, speaks to the need for community-driven, context-specific adaptation of the evidence-proven interventions for cervical cancer prevention, specifically VIA, HPV testing and “see and treat” strategies. While the efficacy and effectiveness of these low-cost strategies have been clearly shown in large, well-conducted trials, there are few implementation studies done in partnership with target communities to adapt and iteratively evaluate the effectiveness of the resulting intervention and implementation strategy. Implementation and dissemination science, or “the scientific study of methods to promote the systematic uptake of research findings and other evidence-based practices into routine practice, and hence, to improve the quality and effectiveness of health services and care,” provides tools to bridge the gap between scientific evidence and public health practices and policy. In addition to the standard clinical effectiveness outcomes, implementation studies evaluate a combination of quantitative, qualitative and process measures to evaluate the feasibility and sustainability of the implementation, essentially exploring and explaining the individual, interpersonal, community and policy-level factors necessary for the success of evidence-based interventions. The above-cited studies of self-collected HPV in Uganda and Kenya are examples of using implementation science research to address the gap between evidence-based cervical cancer prevention, policy and uptake.

## Conclusions

HIV and cervical cancer are intersecting epidemics that disproportionately affect one of the most vulnerable populations in the world: women in LMICs. Despite the biologic, behavioural and demographic overlap, cervical cancer prevention has for the most part been left out of SRH services for HIV-positive women. Similar to the coordinated and multilateral response to the HIV epidemic, an effective programme for cervical cancer prevention among HIV-positive women needs international, national and community leadership for a broad-based and sustainable response. International guidelines for HIV care in LMICs must include a mandate to provide cervical cancer prevention as part of comprehensive SRH care. Funding agencies and local governments must then consider this a key component of HIV care and provide the funding, training, support supervision and accountability necessary to ensure maximal coverage of services. Implementation studies done in partnership with local governments, key stakeholders and programmes providing HIV care will facilitate cervical cancer prevention strategies that are not only included as part of the essential package of services, but are provided in a context-specific way. Cervical cancer prevention has the potential to be effective, sustainable and cost-effective. A crucial part of the implementation strategy will be developing a monitoring and evaluation programme to measure the coverage and quality of cervical cancer prevention services provided as part of comprehensive SRH services for HIV-positive women.

The climate is right for a coordinated response to the dual threat posed by HIV and cervical cancer in LMICs: low-cost strategies, improved health infrastructure and engagement in the healthcare system by a high-risk population. The ability to impact this long-standing global health disparity is well within our reach.
